# Clinical relevance and druggability of sole reciprocal kinase fusions: A large‐scale study

**DOI:** 10.1002/cam4.70191

**Published:** 2024-09-10

**Authors:** Jiao Feng, Tonghui Ma, Chunyang Wang, Baoming Wang, Qian Liu, Zhengchuang Liu, Houquan Tao, Zaiyuan Ye

**Affiliations:** ^1^ General Surgery, Cancer Center Zhejiang Provincial People's Hospital (Affiliated People's Hospital), Hangzhou Medical College Zhejiang China; ^2^ School of Pharmacy, Hangzhou Normal University Zhejiang China; ^3^ Jichenjunchuang Clinical Laboratory Zhejiang China; ^4^ Genecn‐Biotech Co.Ltd Zhejiang China; ^5^ College of Medicine, Zhejiang University Zhejiang China

**Keywords:** NGS, sequencing, sole reciprocal, targeted therapy, tumor

## Abstract

**Background:**

Building on our prior work that RNA alternative splicing modulates the druggability of kinase fusions, this study probes the clinical significance of sole reciprocal fusions. These rare genomic arrangements, despite lacking kinase domains at the DNA level, demonstrated potential RNA‐level druggability in sporadic cases from our prior research.

**Methods:**

Utilizing the large‐scale multicenter approach, we performed RNA sequencing and clinical follow‐up to evaluate a broad spectrum of kinase fusions, including *ALK*, *ROS1*, *RET*, *BRAF*, *NTRK*, *MET*, *NRG1*, and *EGFR*, in 1943 patients.

**Results:**

Our findings revealed 51 instances (2.57%) of sole reciprocal fusions, predominantly in lung (57%), colorectal (14%), and glioma (10%) cancers. Comparative analysis with an MSKCC cohort confirmed the prevalence in diverse cancer types and identified unique fusion partners and chromosomal locales. Cross‐validation through RNA‐NGS and FISH authenticated the existence of functional kinase domains in subsets including *ALK*, *ROS1*, *RET*, and *BRAF*, which correlated with positive clinical responses to targeted kinase inhibitors (KIs). Conversely, fusions involving *EGFR*, *NRG1*, and *NTRK1/2/3* generated nonfunctional transcripts, suggesting the need for alternative therapeutic interventions.

**Conclusion:**

This inaugural multicenter study introduces a novel algorithm for detecting and treating sole reciprocal fusions in advanced cancers, expanding the patient population potentially amenable to KIs.

## 1 BACKGROUND

Oncogenic aberrations due to chromosome translocation and rearrangement, including *ALK*, *ROS1*, *RET*, *BRAF, EGFR*, *and NTRK1/2/3* fusions are important driver variants and therapeutic targets in solid tumor.^1^, ^2^, ^3^, ^4^, ^5^, ^6^ Conventional diagnostic strategies for gene fusions, including immunohistochemistry (IHC) and fluorescence in situ hybridization (FISH) are predefined and low throughput.^7^ Compared to the above mentioned methods, DNA based next generation sequencing (DNA‐NGS) could detect multiple fusions simultaneously and provide more information including structure details of fusion, which own potential medical significance.

Various novel fusions with potential clinical value are being identified based on the DNA‐NGS results. However, the clinical significance of some novel fusions may be misunderstood when the DNA‐NGS result was the only reference. For example, intergenic fusions which contain at least one intergenic junctions, should have present negative outcome at RNA level due to the missing of full‐coding templates based on the DNA‐NGS results. However, previous studies have proved that partial intergenic‐breakpoint fusions could produce functional transcripts and respond to the KIs therapy.^8^, ^9^ In our preliminary studies, we also demonstrated the vital role of RNA alternative splicing in the formation of receptor tyrosine kinase fusions in tumors. For example, specific ALK and ROS1 fusions with abnormal transcripts or frameshift mutations can maintain functional transcription through RNA alternative splicing, which either inserts or deletes nucleotide sequences or even excises specific exons.^10^ Furthermore, for certain rare fusion partners in ALK, ROS1, and RET fusions.^10^, ^11^, ^12^ RNA alternative splicing frequently alters the fusion partner to sustain functional transcription. Additionally, our studies show that RNA alternative splicing manifests tumor‐specific behaviors.^13^ For instance, MET fusions in lung cancer commonly undergo MET 14 skipping due to RNA alternative splicing, a phenomenon not observed in other cancer types like glioma and colorectal cancer. This underlines the complexity and importance of RNA alternative splicing in forming functional fusion transcripts in tumors.

**FIGURE 1:**
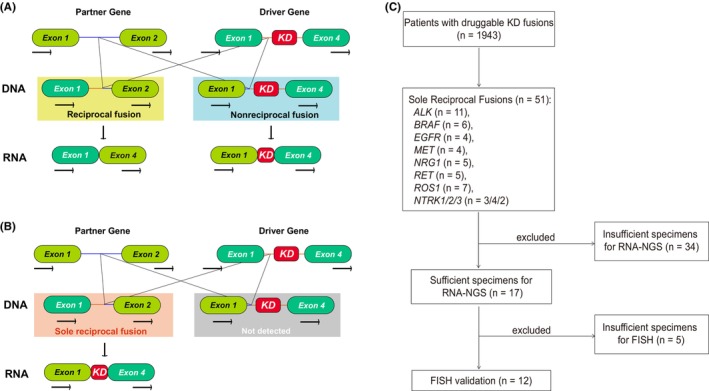
Flow diagram of the study design.

DNA‐NGS can identify various oncogenic fusions constructed by driver genes and partner genes, such as *EML4‐ALK*. Typically, *EML4‐ALK* alone or nonreciprocal/reciprocal *ALK* fusions (*ALK‐EML4* and *EML4‐ALK*) can be successfully sequenced by DNA‐NGS. The intact kinase domain (KD) is retained in *EML4‐ALK* or nonreciprocal/reciprocal ALK fusions. However, sometimes only *ALK‐EML4*, without accompanying *EML4‐ALK*, can be identified at the DNA level, which we refer to as sole reciprocal kinase fusion. The characteristic of sole reciprocal fusions is that the oncogenic kinase genes frequently remain in the opposite position (e.g., 5’ *ALK‐X* 3′) than usual at the DNA level and usually do not have an intact KD (Figure 1A,B).^8^ Sole reciprocal kinase fusion accounted for an substantial proportion in the fusion positive patients, which was around 7% in MSKCC database.^14^ Theoretically, sole reciprocal fusions should not generate functional transcripts due to the lack of kinase activities.^15^ However, our previous cases reported sporadic patients with genomic sole reciprocal fusions had the potential to benefit from the KI therapy as they presented complete kinase structures at RNA level.^16^, ^17^ These cases prompted us to performe a large‐scale multicenter study incorporated a broad spectrum of kinase fusions, such as *ALK*, *ROS1*, *RET*, *BRAF*, *NTRK*, *MET*, *NRG1*, and *EGFR*, and employed RNA sequencing and clinical follow‐up to evaluate the potential clinical value of sole reciprocal fusions. In this study, various oncogenic sole reciprocal fusions identified by DNA‐NGS results were verified by RNA‐NGS and further validated in FISH. We, for the first time, demonstrated that certain sole reciprocal fusions could produce functional transcripts and could benefit from KIs therapy. Our study introduces a novel algorithm for identifying and treating these atypical fusions, thereby widening the patient population potentially responsive to KIs.

## 2 METHODS AND MATERIALS

### 2.1 Patients and samples

Tumor histological specimens (FFPE, formalin‐fixed paraffin‐embedded tissues, or frozen tissues) obtained from solid tumor patients in China between March 2018 and January 2020 were used for NGS detection. All patients were diagnosed via pathological, imaging, and clinical findings.

### 2.2 DNA isolation and targeted sequencing

Genomic DNA was extracted from tumor samples using a QIAamp DNA FFPE Tissue Kit (Qiagen, Valencia, CA, USA) and fragmented to construct a library using KAPA Hyper Prep kits (KAPA, KK8504). DNA fragments of 200 ~ 400 bp were purified by an Agencourt AMPure XP Kit (Beckman Coulter, CA, USA). Fragmented genomic DNA libraries were constructed with the KAPA HTP Library Preparation Kit (Kapa Biosystems, Wilmington, MA, USA) according to the instruction manual. After determining the concentration by a Qubit dsDNA HS Assay kit, DNA libraries were analyzed using Onco PanScan™, which is an 825‐gene panel including major tumor‐related genes, as described previously.^18^

### 2.3 RNA isolation and targeted sequencing

Total RNA was isolated from FFPE samples using the AllPrep DNA/RNA Mini Kit (QIAGEN, Hilden, Germany). The RNA quality and quantity were determined by the Qubit RNA HS Assay (Thermo Fisher Scientific, Waltham, MA, USA) and the 4200 TapeStation System (Thermo Fisher Scientific, Waltham, MA, USA), respectively. Qualified RNA was used to synthesize double‐stranded cDNA using a SuperScript VILO cDNA Synthesis kit (Thermo Fisher Scientific, Waltham, MA, USA) and an Abclonal Second Strand Synthesis Module (Abclonal, Wuhan, China) according to the recommended protocol. To prepare genomic libraries, the double‐stranded cDNA was fragmented and constructed by a KAPA HTP Library Preparation Kit (KAPA Biosystems, Wilmington, MA, USA). The prepared libraries were captured with FusionCapture™, which contains 395 cancer‐related genes, and then subjected to Illumina NovaSeq6000 (Illumina, Inc., San Diego, CA) for paired‐end sequencing as described previously.^18^ The sequencing reads were mapped to UCSC hg19 through HISAT2‐2.0.5,^19^ and FusionMap was used to identify gene fusions.^20^ HISAT (hierarchical indexing for spliced alignment of transcripts) is a highly efficient system for aligning reads from RNA sequencing experiments by using an indexing scheme based on the Burrows‐Wheeler transform and the Ferragina‐manzini (Fm) index, employing two types of indexes for alignment: a whole‐genome Fm index to anchor each alignment and numerous local Fm indexes for very rapid extensions of these alignments. FusionMap, which aligns fusion reads directly to the genome without prior knowledge of potential fusion regions. FusionMap can detect fusion events in both single‐ and paired‐end datasets from either RNA‐Seq or gDNA‐Seq studies and characterize fusion junctions at base‐pair resolution.

### 2.4 FISH

The detection of fusions in *ALK*, *BRAF*, *NTRK1*, *NTRK2*, *RET*, and *ROS1* genes was commissioned to LBP Medicine Science & Technology (Guangzhou, China).

### 2.5 IHC

IHC assay of ALK was carried out by Ventana anti‐ALK (D5F3) rabbit monoclonal primary antibody on FFPE slides, following universal secondary antibody (DAKO). Tumor cells with strong cytoplasmic staining was considered as ALK positive, while without was deemed to be ALK negative.

### 2.6 Public database

The MSK cohort information was obtained from publication: Genomic characterization of metastatic patterns from prospective clinical sequencing of 25,000 patients.^14^

### 2.7 Clinical response evaluation

For patients who received receptor tyrosine kinase inhibitor therapy, clinical responses were evaluated based on computed tomography imaging, according to the Response Evaluation Criteria in Solid Tumors (RECIST) version 1.1.

## 3 RESULTS

### 3.1 Clinical pathological features of sole reciprocal fusions

Of the 1943 cases showed positive for druggable kinase fusions by DNA‐NGS, 51 (2.57%) samples were sole reciprocal fusions (Figure 1C).

Among the patients with sole reciprocal fusions, samples were obtained from 27 men (53%) and 24 women (47%) with ages ranging from 0 to 85 years (average age = 56.8 years, median age = 61 years). Sole reciprocal fusions distributed in various cancer types and the top three diseases involved were lung cancer (57%, 29/51), Colorectal cancer (CRC) (14%, 7/51) and Glioma (10%, 5/51) (Table 1).

**TABLE 1:** Clinicopathologic features of patients with reciprocal fusions.

Clinicopathologic features (*n* = 51)	
Age	
Average Median Range	56.8 years 61 years 0–85 years
Sex	
M F	27 (53%) 24 (47%)
Cancer types	
Lung cancer Colorectal cancer Glioma Fibrous meningioma Gastric cancer Sarcoma Thymic cancer Ovarian cancer Melanoma Prostate cancer Esophageal cancer	29 (57%) 7 (14%) 5 (10%) 1 (2%) 2 (4%) 1 (2%) 1 (2%) 1 (2%) 1 (2%) 2 (4%) 1 (2%)

### 3.2 Sole reciprocal fusions prevalence in various cancers and kinase drivers

**FIGURE 2:**
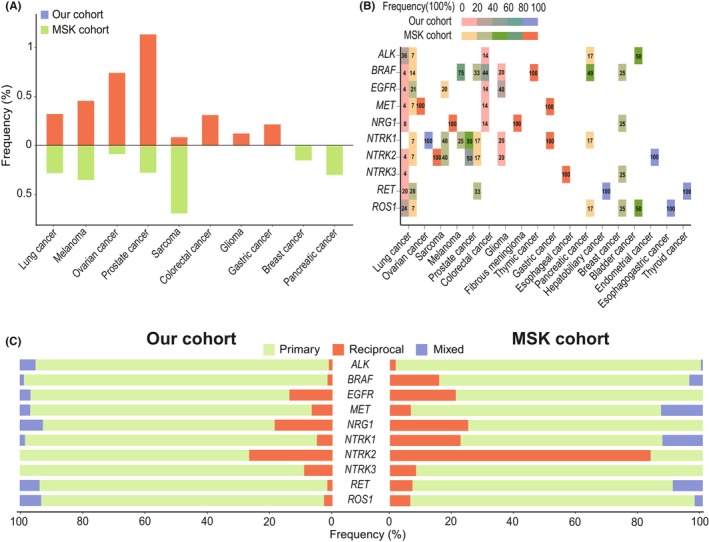
Comparison between our cohort and MSK cohort about sole reciprocal fusions. (A) The incidence of sole reciprocal fusions in typical cancers. (B) Distribution of the proportion of sole reciprocal drivers in each cancer. (C) The distribution of primary, sole reciprocal and mixed fusions in each driver. Primary fusion (driver was in the 3′ end with only one fusion partner, like 5′ X‐ALK 3′), Sole reciprocal fusion (driver was in the 5′ end, like 5’ ALK‐X 3′) and Mixed fusion (driver was in the 3′ end with more than 2 fusion partners or drivers co‐occurred in the 5′ and 3′ end, like 5′ X‐ALK 3′ and 5’ ALK‐Y 3′).

Significant amount of sole reciprocal fusions were identified in multiple cancers in our cohort and the similar result was also found in MSK cohort. In our and MSK cohort, sole reciprocal fusions with different proportions were found in lung cancer (0.32%, 29/8668 vs. 0.28%, 14/5024), melanoma (0.46%, 1/218 vs. 0.35%, 4/1142), ovarian cancer (0.74%, 1/135 vs. 0.08%, 1/1183), prostate cancer (1.14%, 2/176 vs. 0.28%, 6/2172) and sarcoma (0.09%, 1/1168 vs. 0.69%, 5/722) (Figure 2A). Moreover, numerous oncogenic drivers (*ALK, BRAF, EGFR, MET, NRG1, NTRK1/2/3, RET*, and *ROS1*) involved in sole reciprocal fusions were found in those cancers (Figure 2B). Lung cancer present the highest diversity of driver types in both cohorts and *ALK* (36%, 10/28) and *RET* (28%, 4/14) sole reciprocal fusions were the top driver genes in our and MSK cohort, respectively. Additionally, sole reciprocal fusions represented a nonnegligible portion of the whole kinase fusions, particularly in some specific drivers, as *NTRK2* sole reciprocal fusions accounted for up to 25% and 83% of all *NTRK2* fusions in our and MSK cohort, respectively (Figure 2C).

### 3.3 Molecular characteristics of sole reciprocal fusions

**FIGURE 3:**
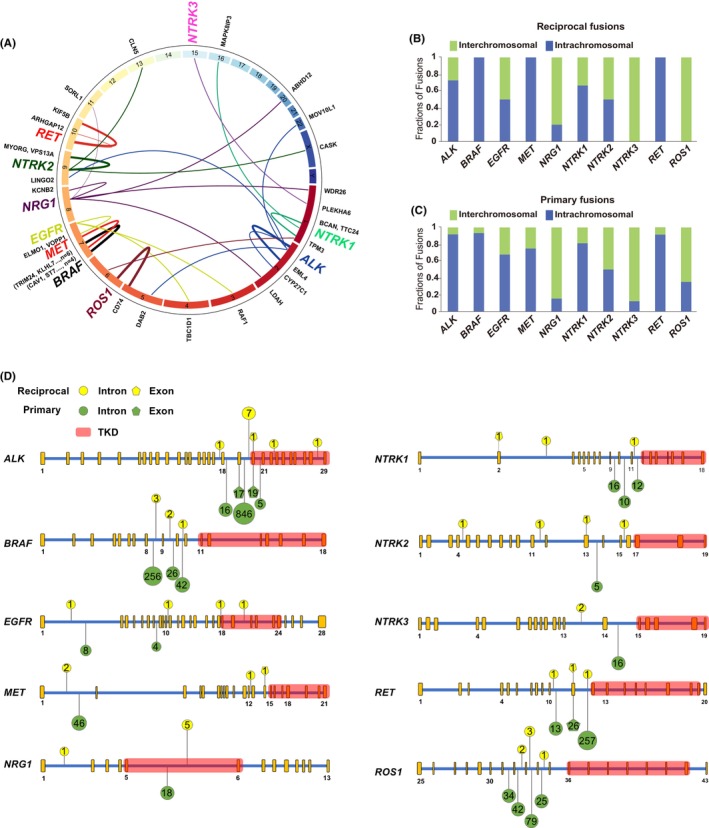
The molecular characteristics of sole reciprocal fusions in our cohort. (A) Circos plot of chromosomal translocations in our cohort. (B) Chromosomal distribution pattern of sole reciprocal fusions in each driver. (C) Chromosomal distribution pattern of primary fusions in each driver. (D) The distribution of breakpoints in each driver between primary and sole reciprocal fusions.

Sole reciprocal fusions with multiple partners were identified in each oncogenic driver (Figure 3A). The diversity of fusion partners bias could be found in drivers. For example, in the *ALK* fusions, *EML4* is the dominant partner (64%, 7/11). However, no same sole reciprocal fusion was found in *EGFR* (*n* = 4), *MET* (*n* = 4), *NRG1* (*n* = 5) and *NRTK* (*n* = 7) (Figure 3A). Similarly, MSK cohort also displayed different distribution pattern of sole reciprocal fusions in various drivers (Figure S1A). Further, both our and MSK's study showed that the chromosomal distributions of sole reciprocal fusions varied in different drivers (Figure 3B; S1B). In both cohorts, *BRAF*, *MET* and *RET* sole reciprocal fusions mainly rearranged in the intra‐chromosome. On the contrary, high proportion of inter‐chromosomal rearrangements were found in the *EGFR, NRG1* and *NTRK3* (Figure 3B; S2). Interestingly, although the chromosomal distribution of sole reciprocal fusions in specific driver gene is unique, sole reciprocal fusions and primary fusion of the same gene generally exhibit similar chromosomal distribution characteristics (Figure 3C).

Also, fusion breakpoints analysis showed that the breakpoints distribution between sole reciprocal and primary fusions were different in some drivers (Figure 3D;S1D), including *ALK, EGFR, MET*, *and NTRK1/2/3*. For example, the breakpoints of primary fusions in *MET* concentrated in intron 1 where was also the breakpoints peak in the sole reciprocal fusions. Apart from that, intron 12 and exon 14 were the distinctive breakpoints in sole reciprocal fusions of *MET*. Remarkably, the breakpoints distribution of *NTRK2* and *NTRK3* were totally different between reciprocal and primary fusions (Figure 3D). The similar trend was also found in MSK cohort (Figure S1D). Additionally, different from the primary fusions whose breakpoints concentrated in the adjacent areas, the breakpoints of sole reciprocal fusions distributed in a scattered pattern. As the breakpoints in *ALK* primary fusions frequently displayed in intron 18–20, while dispersedly distributed in intron 17, intron 19, intron 22 and intron 28 in the sole reciprocal form.

### 3.4 Cross‐validation of sole reciprocal fusions

**FIGURE 4:**
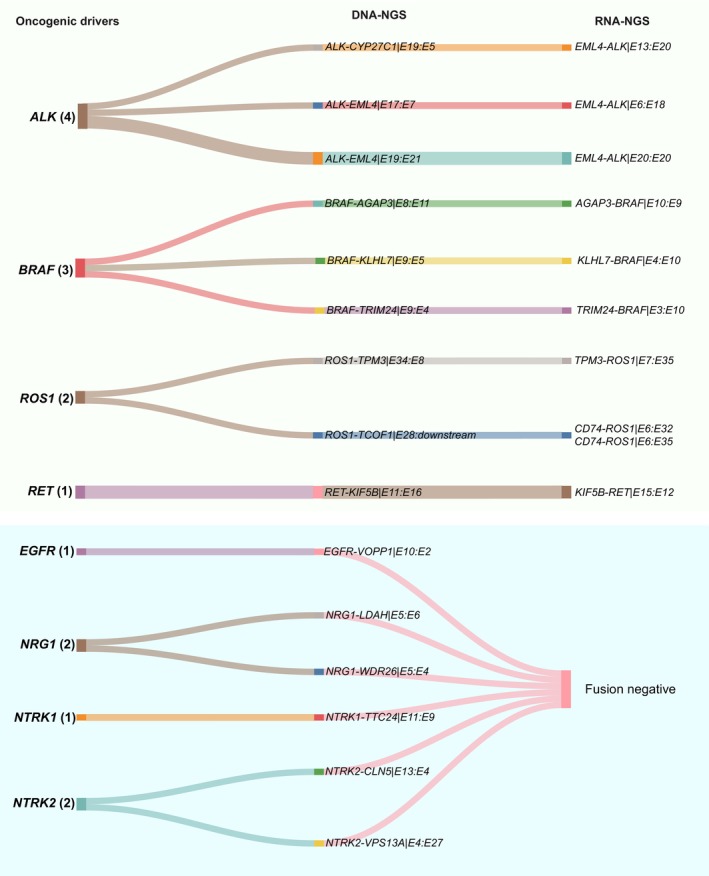
Cross‐validation of sole reciprocal fusions with RNA‐NGS.

Seventeen samples with sole reciprocal fusions were successfully performed on RNA‐NGS, and functional fusion transcripts with complete KD were verified in 11 out of these samples (Figure 4). Among which, canonical *EML4‐ALK* fusion transcripts were present in all four samples carrying sole reciprocal *ALK* fusion by RNA‐NGS, and further confirmed by FISH (Table S1). Similarly, chimeric fusion transcripts with intact KD were confirmed in samples with sole reciprocal fusion formed by *BRAF*, *RET* and *ROS1* fusion and validated by FISH (Table S1). On the contrary, samples with sole reciprocal fusions formed by *EGFR*, *NTRK1/2*, or *NRG1* did not generate functional fusion transcripts by RNA‐NGS (Table S1).

### 3.5 Targeted treatment and clinical efficacy of single sole reciprocal fusions

**FIGURE 5:**
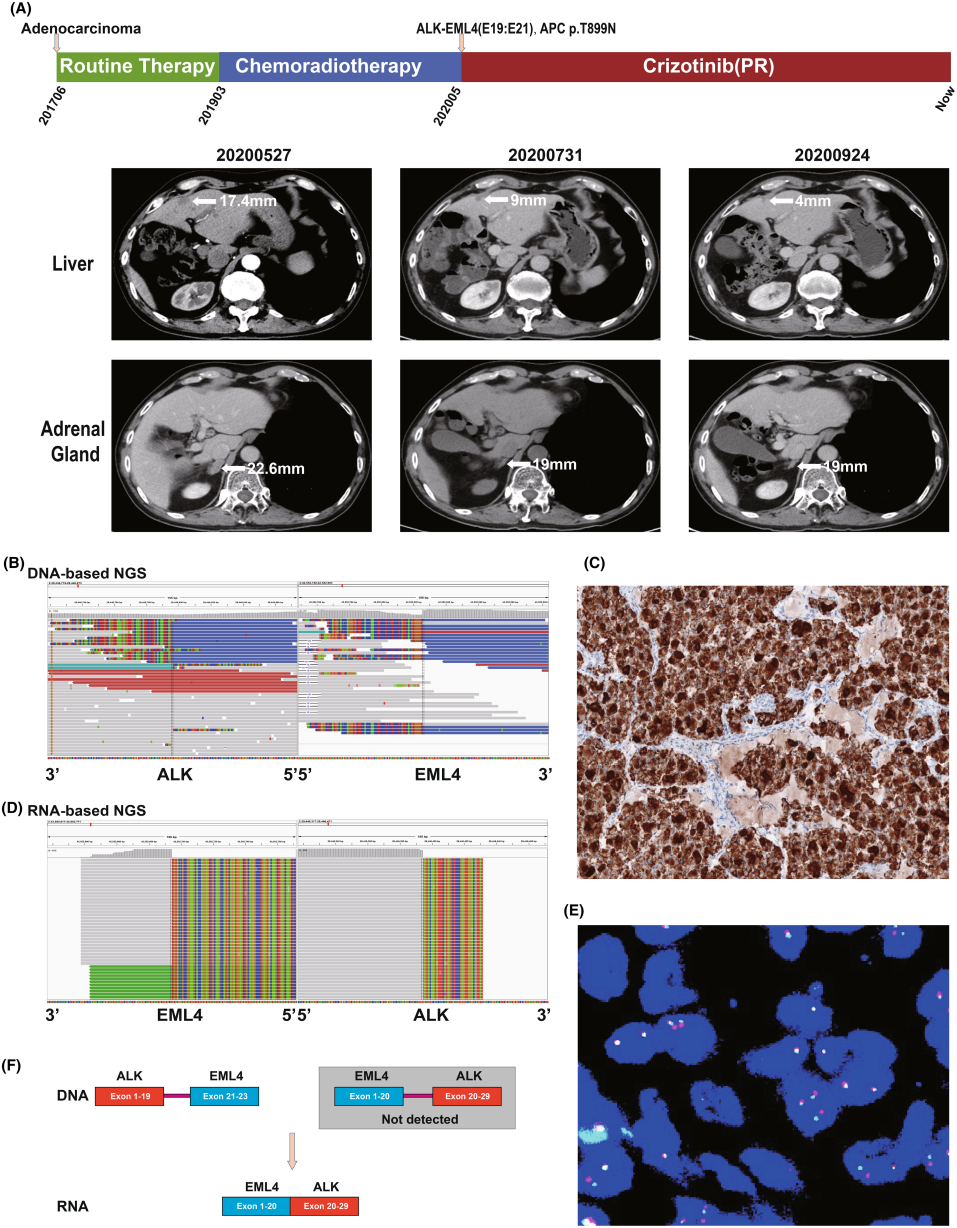
Treatment diagram and sequence analysis of the ALK fusion. (A) Treatment course and representative images of CT scans of liver and adrenal gland from the patient before and after therapy. (B) Assembled fusion reads at DNA level were shown by the Integrative Genomics Viewer. (C) Immunohistochemistry analysis of ALK fusion. (D) RNA sequencing data were visualized in Integrative Genomics Viewer. (E) The split signals were detected by FISH. (F) Possible schematic diagram of ALK‐EML4 detected by DNA‐based NGS, but EML4‐ALK fusion was identified by RNA‐based NGS.

Patients with sole reciprocal fusions were followed up. Based on the medical guideline and patients' strong willing, four lung adenocarcinoma patients (case 1, 3, 5, 51) with sole reciprocal fusions were treated with inhibitors (Crizotinib or Alectinib), and all showed partial response (PR) (Table 2). As one example shown in Figure 5, case 5 was a 65 year old male with only a sole reciprocal fusion of *ALK‐EML4* identified by DNA‐NGS (Figure 5A,B). IHC were perfomed on sample of this patient to verify the possibility of *ALK* fusion, and crizotinib was administered based on the positive result of ALK protein staining (Figure 5C). CT scan revealed a significant decrease in both liver metastatic focus (17.4 mm–4 mm) and adrenal gland metastatic focus (22.6 mm–19 mm) after 16 weeks of treatment. PR has been achieved for over 30 months during manuscript preparation (Figure 5A). In further detection, canonical *EML4‐ALK* presented at transcriptional level (Figure 5D) and positive *ALK* signals were obtained from FISH results (Figure 5E). Case 1, case 3, and case 51 with single sole reciprocal fusions were also administered with inhibitors after getting positive result from IHC or FISH and PR was achieved. Those cases indicated that in patients with sole reciprocal fusions, canonical *ALK* or *ROS1* fusions are potentially present and could benefit from inhibitors treatment (Figure 5F).

**TABLE 2:** The treatment diagram of patient with ALK sole reciprocal fusions.

Case	DNA‐NGS	FISH	RNA‐NGS	IHC	KIs	Optimal Response
1	*ALK‐CYP27C1*|E19:E5	+	*EML4‐ALK*|E13:E20	+	Crizotinib	PR
3	*ALK‐EML4*|E19:E21	+	*EML4‐ALK*|E20:E20	+	Alectinib	PR
5	*ALK‐EML4*|E19:E21	+	*EML4‐ALK*|E20:E20	+	Crizotinib	PR
51	*ROS1‐TCOF1|E28:downstream*	+	*CD74‐ROS1|E6:E32* *CD74‐ROS1|E6:E35*	+	Crizotinib	PR

Abbreviations: FISH, fluorescence in situ hybridization; IHC, immunohistochemistry; NGS, next‐generation sequencing, PR, Partial response.

### 3.6 Proposed algorithm for sole reciprocal fusions detection in advanced cancers

**FIGURE 6:**
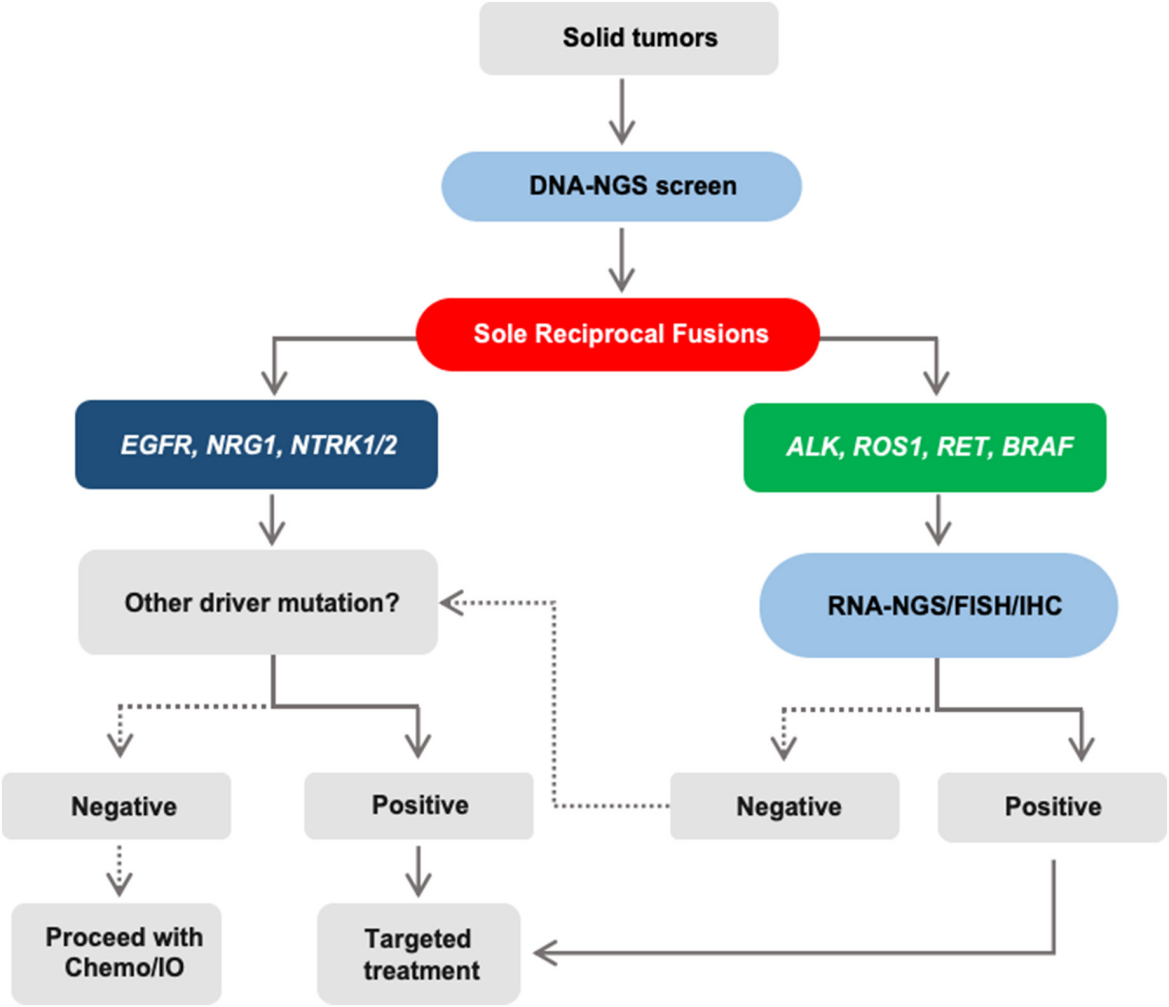
Proposed algorithm for sole reciprocal fusions detection in advanced cancers.

Based on the results of our study, significant amount of sole reciprocal fusions were functional. Additionally, the genetic bias was observed in different sole reciprocal fusions. Results indicated the ones formed by *ALK*, *RET*, *ROS1*, and *BRAF* could be indicators of functional fusion transcripts. Therefore, once sole reciprocal fusions with *ALK*, *RET*, *ROS1*, and *BRAF* were identified, the cross‐validation methods, such as RNA‐NGS and IHC, were recommended to confirm the functional transcript. On the contrary, patients with sole reciprocal fusions with *EGFR, NRG1*, and *NTRK1/2* at genomic level should explore alternative therapy options, such as other druggable mutations (Figure 6).

## 4 DISCUSSION

With the ability of detecting oncogenic fusions and identifying beneficial populations, DNA‐NGS has been widely adopted and numerous novel fusions with medical potential had been discovered.^21^ sole reciprocal fusions, as a novel fusion type, had been sporadically mentioned in the previous studies.^8^, ^22^ However, the clinical value of sole reciprocal fusions remains to be confirmed. Here, for the first time, we reported that partial sole reciprocal fusions (*ALK*, *BRAF*, *RET*, and *ROS1*) identified by DNA‐NGS could produce the functional transcripts. Furthermore, the patients with sole reciprocal fusions were benefited from KIs therapy. Additionally, an algorithm for sole reciprocal fusions detection in advanced cancers was proposed.

Fusions with KD have been widely proved to be clinically relevant and plenty of patients benefited from the KIs therapy. Compared to the primary fusions with intact KD, sole reciprocal fusions is a rare type of oncogenic rearrangement which usually presented with incomplete KD at genomic level. Theoretically, sole reciprocal fusions without full‐coding KD should not benefit from the targeted KIs' therapy and is often overlooked in clinical practice. However, our previous studies reported sporadic sole reciprocal fusions could present intact KDs at RNA level and patients obtained response to targeted agents.^16^, ^17^ Those findings emphasized the potential clinical value of sole reciprocal fusions and systemic analysis of those fusions were essential.

The sole reciprocal fusions were found to be prevalent in multiple cancers in our and MSK cohorts. Among those cancers, the dominant diseases in both cohorts were prostate cancer and sarcoma, which owned less therapy targets.^23^, ^24^ Furthermore, sole reciprocal fusions were identified in various oncogenic drivers with nonnegligible proportion. Among them, multiple targeted drugs have been approved in *ALK*, *ROS1*, *RET*, and *NTRK* fusions by FDA. Therefore, the precise identification of sole reciprocal fusions by the high‐throughput sequencing technology (NGS) are important and meaningful to cancer patients. While, considering the cost‐effectiveness, commercial DNA‐NGS panel usually only cover hotspot introns of primary fusions. Previous studies and our results showed primary fusions constituted top proportion in the oncogenic rearrangements and its breakpoints in the drivers are relatively intensed. For example, 90% breakpoints in *ALK* primary fusions distributed in intron 18 and intron 19.^25^ Similar result also could be found in the intron 1 of the *MET* primary fusions.^13^ However, different from the primary fusions, the breakpoints distribution in sole reciprocal fusions are more diverse and a high proportion of breakpoints distributed in nonhotspot introns. Those results indicated that the scale of sole reciprocal fusions were inevitablely underestimated when DNA‐NGS panels were routinely utilized in clinical practice, although some nonhotspot introns had been covered in our and MSK panel.

The clinical significance of sole reciprocal fusions was further explored and the benefits varied with genes. sole reciprocal fusions of *ALK*, *BRAF*, *RET*, and *ROS1* were found to be functional at transcript level. Furthermore, four patients with genomic sole reciprocal *ALK* and *ROS1* fusions benefited from targeted KI therapy. Those results demonstrated the potential clinical value of sole reciprocal *ALK*, *BRAF*, *RET*, and *ROS1* fusions. On the contrary, the functional transcript of *EGFR*, *NRG1*, and *NTRK1/2* fusions could not be detected. Clinical relevance of sole reciprocal fusions formed by *MET* or *NTRK3* were still unclear due to the limited sample size in current study. A larger and comprehensive study for individual fusion event would provide clarification on whether a presence of sole reciprocal fusions formed by *ALK, BRAF*, *RET*, *ROS1*, or others could be used as direct evidence for clinical decisions such as targeted therapies.

The mechanism about inconsistency of sole reciprocal fusions between DNA and RNA was still unknown. Previous study demonstrated that the actual fusion partner of intergenic fusions initially attached to an intergenic area, the deletion of intergenic region during transcription resulted in the fusion of actual fusion partner to the kinase driver gene. Similarly, the real partners of sole reciprocal fusions may hide in the intergenic region at genomic level, which could transcribe and fuse with drivers during transcription. Additionally, the chromothripsis or complex genomic rearrangement splicing might be another explanation of the inconsistency as well.^26^ The discrepant results between DNA‐NGS and RNA‐NGS may also attribute to the sequencing depth.^27^ However, there were still no fusions with completed KD detected at depth of more than 500X in our study. Therefore, the exact mechanism still need to be clarified in the future.

In summary, our result showed that sole reciprocal fusion is a rare but significant potential druggable target for cancer treatment. Genetic bias was observed, in particular the ones formed by *ALK, RET, ROS1*, and *BRAF* were indicators of functional fusion transcripts. Monitoring the above‐mentioned sole reciprocal fusions may be directly applicable to clinical practice. In total, extra 1.7‰ patients with solid tumor which won't be recommended to the KIs therapy before could be included in targeted therapy. This breakthrough shall encourage more studies on validating consistence between single sole reciprocal fusions and functional fusion transcripts in a larger scale and ultimately maximize the understanding of NGS data for improved accuracy of molecular diagnosis.

## 5 LIMITATION

Limited number of samples was included in the study due to the low percentage of the sole reciprocal fusions in the real world. Additionally, only one third samples were sequenced by RNA‐NGS because of the limited specimen. Also, the proportion of sole reciprocal fusions possibly under‐estimated because not all introns were covered by DNA‐NGS.

## AUTHOR CONTRIBUTIONS

**Jiao Feng:** Investigation (equal); visualization (equal); writing – original draft (equal). **Tonghui Ma:** Supervision (equal). **Chunyang Wang:** Conceptualization (equal); formal analysis (equal). **Baoming Wang:** Writing – original draft (equal). **Zhengchuang Liu:** Resources (equal). **Qian Liu:** Methodology (equal). **Houquan Tao:** Supervision (equal); validation (equal). **Zaiyuan Ye:** Methodology (equal); project administration (equal).

## FUNDING INFORMATION

This work was supported by the Zhejiang Province Medical Science and Technology Projects grant No. 2016KYB027 and Grant No. 2017KY010.

## CONFLICT OF INTEREST STATEMENT

There is no interest conflict. THM, CYW and BMW were employed by Jichenjunchuang Clinical Laboratory and Genecn‐Biotech Co.Ltd.

## ETHICAL APPROVAL AND CONSENT TO PARTICIPATE

The study was approved by the Institutional Review Board of Zhejiang Provincial People's Hospital (2021QT420), and individual consent for this retrospective analysis was waived, patients below 18 years old were provided with signed informed consent by their legal parents/legal guardians for the research use of clinical data and publication.

## Supporting information


Data S1.

## Data Availability

The raw sequence data reported in this paper have been deposited in the Genome Sequence Archive in National Genomics Data Center, China National Center for Bioinformation / Beijing Institute of Genomics, Chinese Academy of Sciences (GSA‐Human: HRA007923) that are publicly accessible at https://ngdc.cncb.ac.cn/gsa‐human.
