# Supplementary material for: Clinical relevance and druggability of sole reciprocal kinase fusions: A large‐scale study
Source: Cancer Med. 2024 Sep 10;13(17):e70191. doi: 10.1002/cam4.70191 (PMC11386300; doi:10.1002/cam4.70191)
Supplement: Supplementary file 1 — Data S1. [file CAM4-13-e70191-s001.docx]

**Table S1. Fusion partners and breakpoints of sole reciprocla fusions identified by DNA-NGS, RNA-NGS and FISH**

| **Case** | **ID** | **Sex** | **Age** | **Cancer** | **DNA-NGS** | | | | **RNA-NGS** | **FISH** |
| --- | --- | --- | --- | --- | --- | --- | --- | --- | --- | --- |
|  |  |  |  |  | **Fusion** | **Broke Position** | **Vaf** | **Effective sequencing depth** | **Fusion** |  |
| 1 | P1910180045 | F | 48 | LC | *ALK-CYP27C1*\|E19:E5 | Chr2:29446807-Chr2:127956210 | 18.80% | 3901.78 | *EML4-ALK*\|E13:E20 | + |
| 3 | P2001100053 | F | 55 | LC | *ALK-EML4*\|E19:E21 | Chr2:29446768-Chr2:42553273 | 21.80% | 1563.89 | *EML4-ALK*\|E20:E20 | + |
| 4 | P2003140022 | F | 34 | LC | *ALK-EML4*\|E17:E7 | Chr2:29450394-Chr2:42502778 | 4.85% | 1407.87 | *EML4-ALK*\|E6:E18 | N/A |
| 5 | P1907100028 | M | 65 | LC | *ALK-EML4*\|E19:E21 | Chr2:29446796-Chr2:42552827 | 8.70% | 968.45 | *EML4-ALK*\|E20:E20 | + |
| 12 | P1907300012 | F | 54 | BC | *BRAF-AGAP3*\|E8:E11 | Chr7:140491444-Chr7:150830297 | 5.71% | 1758.87 | *AGAP3-BRAF*\|E10:E9 | + |
| 13 | P2003100022 | F | 35 | G | *BRAF-KLHL7*\|E9:E5 | Chr7:140487210-Chr7:23174387 | 28.16% | 1965.71 | *KLHL7-BRAF*\|E4:E10 | + |
| 15 | P1908200005 | M | 85 | BC | *BRAF-TRIM24*\|E9:E4 | Chr7:140487251-Chr7:138202758 | 9.86% | 2210.37 | *TRIM24-BRAF*\|E3:E10 | N/A |
| 21 | P2010130063 | M | 34 | BC | *EGFR-VOPP1*\|E10:E2 | Chr7:55225169-Chr7:55602526 | 9.70% | 2188.36 | - | N/A* |
| 28 | P2103280201 | F | 46 | FM | *NRG1-LDAH*\|E5:E6 | Chr8:32512177-Chr2:20910503 | 12.36% | 1818.44 | - | N/A* |
| 30 | P1908240008 | M | 56 | LC | *NRG1-WDR26*\|E5:E4 | Chr8:32483963-Chr1:224613813 | 7.31% | 1759.53 | - | N/A |
| 33 | P1912240099 | M | 68 | GC | *NTRK1-TTC24*\|E11:E9 | Chr1:156844853-Chr1:156555047 | 7.13% | 1464.1 | - | - |
| 35 | P1912200058 | M | 49 | LC | *NTRK2-CLN5*\|E13:E4 | Chr9:87359923-Chr13:77574609 | 6.98% | 1712.74 | - | - |
| 37 | P1911050054 | M | 74 | S | *NTRK2-VPS13A*\|E4:E27 | Chr9:87302080-Chr9:79892933 | 18.45% | 1752.15 | - | - |
| 43 | P1905090064 | F | 49 | LC | *RET-KIF5B*\|E11:E16 | Chr10:43610118-Chr10:32316358 | 5.48% | 885.75 | *KIF5B-RET*\|E15:E12 | + |
| 44 | P2005150067 | M | 45 | LC | *RET-KIF5B*\|E11:E16 | Chr10:43610193-Chr10:32312053 | 8.46% | 2277.46 | *KIF5B-RET*\|E15:E12 | + |
| 50 | P2004040063 | M | 65 | LC | *ROS1-TPM3*\|E34:E8 | Chr6:117645219-Chr1:154140931 | 0.98% | 2682.53 | *TPM3-ROS1*\|E7:E35 | + |
| 51 | P2108180247 | F | 44 | LC | *ROS1-TCOF1\|E28:downstream* | chr6:117663476-chr5:149782946 | 5.3% | 1050.34 | *CD74-ROS1\|E6:E32*  *CD74-ROS1\|E6:E35* | + |

FISH: fluorescence in situ hybridization; NGS: next-generation sequencing; M, Male; F, Female; LC, Lung Cancer; BC, Bowel Cancer; G, Glioma; FM, Fibrous meningioma; GC, Gastric Cancer; S, Sarcoma; E, Exon; N/A, Not Available; N/A*, FISH Probe Not Available;


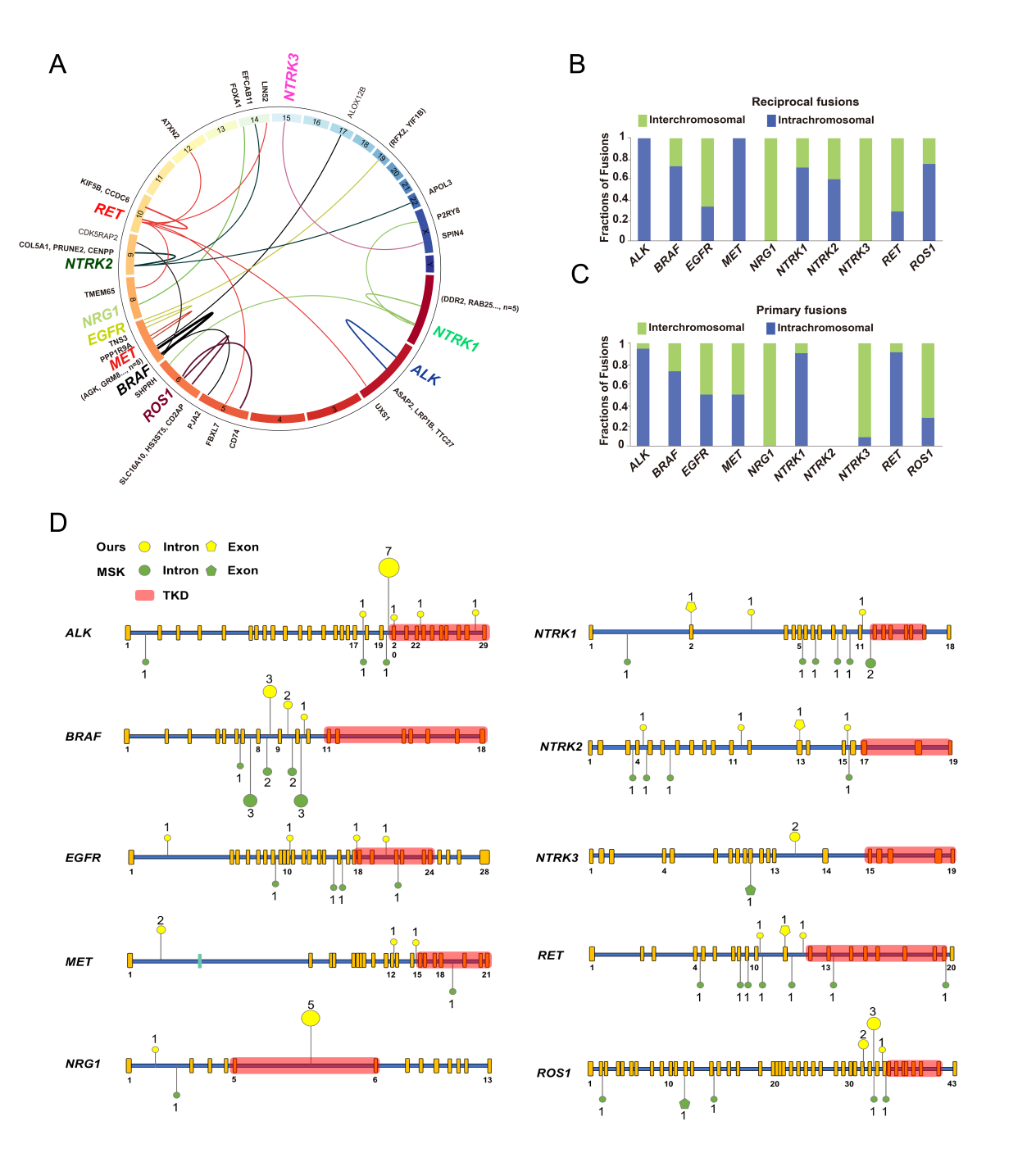


Fig S1. The molecular characteristics of sole reciprocal fusions. A, Circos plot of chromosomal translocations in our cohort. B, Chromosomal distribution pattern of sole reciprocal fusions in each driver. C, The distribution of breakpoints in each driver between primary and sole reciprocal fusions.
